# Responsibilities with conflicting priorities: a qualitative study of ACT providers’ experiences with community treatment orders

**DOI:** 10.1186/s12913-018-3097-7

**Published:** 2018-04-18

**Authors:** Hanne Kilen Stuen, Anne Landheim, Jorun Rugkåsa, Rolf Wynn

**Affiliations:** 1 Norwegian National Advisory Unit on Concurrent Substance Abuse and Mental Health Disorders, Innlandet Hospital Trust, Brummundal, Norway; 2 Department of Clinical Medicine, Faculty of Health Sciences, UiT – The Arctic University of Norway, Tromsø, Norway; 3 Norwegian Centre for Addiction Research, University of Oslo, Oslo, Norway; 4 Health Services Research Unit, Akershus University Hospital, Lørenskog, Norway; 5 Centre for Care Research, University College of Southeast Norway, Porsgrunn, Norway; 6 Divison of Mental Health and Addictions, University Hospital of North Norway, Tromsø, Norway

**Keywords:** Assertive community treatment, Community treatment order, Engagement strategies, Provider experiences, Compulsory medication, Coercion

## Abstract

**Background:**

Patients with severe mental illness may be subjected to Community Treatment Orders (CTOs) in order to secure that the patients adhere to treatment. Few studies have investigated the use of CTOs within an Assertive Community Treatment (ACT) setting, and little is known about how the tension between the patients’ autonomy and the clinicians’ responsibility to act in the patients’ best interest are resolved in practice. The aim of this study was to explore the service providers’ experiences with CTOs within an ACT setting.

**Methods:**

The study was based on reviews of case files of 15 patients, eight individual qualitative in depth interviews and four focus group interviews with service providers involved in ACT and decisions related to CTOs. A modified grounded theory approach was used to analyze the data.

**Results:**

The main theme ‘responsibility with conflicting priorities’ emerged from data analysis (case file reviews, individual interviews and focus group interviews). The balance between coercive approaches and the emphasis on promoting patient autonomy was seen as problematic. The participants saw few alternatives to CTOs as long-term measures to secure ongoing treatment for some of the patients. However, participants perceived the ACT model’s comprehensive scope as an opportunity to build rapport with patients and thereby better meet their needs. The team approach, the ACT providers’ commitment to establish supportive relationships and the frequent meetings with patients in their home environment were highlighted. The ACT approach gave them insight into patients’ everyday lives and, in some cases a greater sense of security when considering whether to take patients off CTOs.

**Conclusions:**

Many of the participants viewed CTOs as helpful in securing long-term treatment for patients. CTO decision-making was described as challenging and complex and presented the providers with many dilemmas. The ACT approach was considered as helpful in that it afforded comprehensive, patient-centered support and opportunities to build rapport.

**Electronic supplementary material:**

The online version of this article (10.1186/s12913-018-3097-7) contains supplementary material, which is available to authorized users.

## Background

Despite a lack of clear evidence of effectiveness, more than 75 jurisdictions worldwide have implemented community treatment orders (CTOs), which is a legal mechanism to secure the treatment adherence of patients with severe mental illness living in the community [1]. Besides mandating patients to adhere to treatment, the CTO regime also allows for a rapid recall to hospital for its enforcement. In Norway, CTOs have been part of the Mental Health Care Act (MHA) [2] since 1961. Despite being debated in many countries, there has been surprisingly little discussion about their use in Norway [3].

In Norway, the involuntary admission rates and the CTO rates are relatively high compared to other western countries and reducing the use of coercion has for many years been a central policy goal [4]. Although two national action plans have been implemented, the involuntary admission rates and the geographical differences in use within Norway have been remarkably stable, while the use of CTOs actually seems to increase [5, 6]. While we lack reliable knowledge about the extent of CTOs, it is estimated that more than one of three patients are placed under CTOs following involuntary hospital admissions [6].

The use of coercion in the Norwegian MHA is based on clinical judgment [7–9]. The legal criteria for involuntary inpatient admissions and CTOs are identical [2]. In addition to severe mental illness (such as psychosis), one of two criteria must be met; the treatment criterion or the danger criterion. The treatment criterion states that ‘…the application of compulsory mental health care must be seen as necessary to prevent the person concerned from having the prospects of his or her health being restored or significantly improved considerably reduced, or it is highly probable that the condition of the person concerned will significantly deteriorate in the very near future’ [2]. The danger criterion states that admission is needed because the patient represents an ‘… obvious and serious risk to his or her own life and health or those of others on account of his or her mental disorder’ [2]. It is also required that voluntary treatment has been tried and the CTO must be considered as the best solution for the patient. Recently (after the current study was carried out), a capacity criterion has been introduced in the Norwegian MHA [2]. Psychiatrists and clinical psychologists (Responsible Clinicians or RCs) in the specialized mental health services are authorised to make CTO decisions, and are required to conduct clinical assessments every third month. Patients on CTOs typically receive primary health care follow- up services in the municipality and are required to attend office-based assessments at the Community Mental Health Centre (CMHC). Local Supervisory Commissions (SCs) are responsible for safeguarding patients’ legal rights to ensure the criteria are still met. To compel patients placed on a CTO to take medication, a separate compulsory medication order (CMO) must be in place.

Studies in Norway and internationally show that psychiatrists and patients’ families typically are more positive to coercive practices in general and CTOs than patients, who often have more mixed views [10–19]. Even if patients describe CTOs as less coercive than hospital admissions, many patients dislike having to take medications and experience that they have little influence on treatment decisions [16–19]. Studies show that health professionals value the CTO scheme for providing security and structure after discharge from hospital [14, 15, 20–23]. Health professionals often describe CTOs as a useful tool to facilitate stable medication and follow-up because of the patients’ lack of insight [23, 24]. Although the CTO scheme has been used for more than fifty years in Norway, only two published studies of health professionals’ experiences with patients on CTOs exists, to our knowledge. Stensrud et al. [23] found that even if the patients were stable over time, the RCs were worried about relapse and therefore reluctant to discontinue the CTOs. Riley et al. [22], from a different geographical region, show that the organization of the local health care seems to influence on the decision-makers’ role and the implementation of the CTO.

Results from three RCTs and meta-analyses on the effectiveness of CTOs show no clear difference in service use, social functioning, mental state or quality of life compared to standard care [1, 25–28]. Some studies show that CTOs, combined with assertive community treatment (ACT) or intensive case management, were associated with positive outcomes such as decreased hospitalization, increased medication possession, reduced violence, and even increased subjective quality of life [29–35]. However, possible selection effects have been pointed out [1].

In a report from 2008, it was estimated that more than 4000 persons with severe mental illness in Norway (approximately 1/1000) did not receive appropriate mental health services [36]. In 2009, the Norwegian health authorities decided to fund the piloting of ACT teams in order to provide effective treatment and rehabilitation to people with severe mental illness who have not engaged with traditional services. In Norway there has been no tradition for using intensive case management. International research suggests that ACT is successful in engaging service users with a history of treatment and service use discontinuity, reducing hospitalization, increasing housing stability, and reducing homelessness [37, 38]. ACT provides in-vivo services, includes a multidisciplinary approach, shared caseload, responsibility for health and social care, time-unlimited treatment, assertive engagement techniques and a high frequency of contacts [37, 39, 40]. Even if the ACT model did not initially describe the use of coercive interventions, later model revisions allow for the time-limited use of restrictive interventions and formal coercion when necessary to promote safe community living [40].

In the 12 first Norwegian ACT teams that were established in the period between December 2009 and February 2011, a total of 38% of the patients were subjected to CTOs at enrolment into ACT during the teams’ first year of operation [41]. Few studies have investigated the use of CTOs within an ACT setting, and little is known about how the tension between the patients’ autonomy and the clinicians’ responsibility to act in the patients’ best interest are resolved in practice. The aim in this qualitative study was to explore the RC’s and ACT providers’ experiences with making decisions about CTOs within an ACT setting.

## Methods

### Recruitment and sample

This qualitative study is part of the national evaluation of the 12 first Norwegian ACT teams [42]. There was significant variation across the ACT teams in CTO rates; between 10% and 59% of the patients were subject to CTOs at intake during the teams’ first year of operation. While the ACT psychiatrist or clinical psychologist was responsible for conducting regular CTO assessments in most of the teams, some teams had deferred this responsibility to the CMHCs. For the present study we chose teams with different CTO rates and ways of organizing the responsibility for CTOs, to broaden the range of included experiences [43]. All the teams had been in place at least 2,5 years before the study started. An interview study with 15 patients has already been published [44]. An overview of the four included ACT teams’ CTO rates and the organization of the CTO responsibility are presented in Table 1.

**Table 1:** Overview of CTO responsibilities in the four included ACT teams (at 30 months)

Team number	Patients in Team	Patients on CTOs (%)	Responsible for CTOs	Responsible for CMOs
Team 1	76	40 (52)	ACT psychiatrist	ACT psychiatrist
Team 2	67	4 (6)	ACT psychologist	ACT psychiatrist
Team 3	68	23 (33)	CMHC psychologist	ACT psychiatrist
Team 4	38	13 (34)	CMHC psychologist	CMHC psychiatrist

### Participants

The sample consisted of eight RCs (six ACT psychiatrists and two CMHCs clinical psychologists) who participated in individual interviews and 20 ACT providers who participated in focus group interviews. The psychologists at the CMHC and the ACT psychiatrists were all invited to discuss their experiences with CTOs within an ACT setting. In connection with the patient study [44], we asked the participating patients for permission to read their case files and to talk with the ACT providers and the RCs about their case.

We conducted focus group interviews with 20 ACT providers from the four ACT teams one year after the individual interviews with the RCs. The purpose of having separate interviews with the RCs and to conduct focus-group interviews with ACT providers was to compare and contrast the ACT providers’ and the RCs’ different roles and responsibilities regarding CTO use. For the focus group interviews, recruitment was aimed at ACT providers with at least two years’ experience with ACT. Many ACT providers and RCs would have had much longer experience with managing patients in the community on CTOs. Because we knew that some teams had struggled with recruiting psychiatrists, we did not use other selection criteria than that they had been responsible for the CTO case for one of the 15 patients interviewed in Study 1. An overview of the participants can be seen in Table 2.

**Table 2:** Participants in individual interviews and focus group (FG) interviews

	Individual interviews	FG 1 (Team 1)	FG 2 (Team 2)	FG 3 (Team 3)	FG 4 (Team 4)
RCs					
Psychiatrists	6				
CMHC psychologist	2				
ACT providers					
Psychologists			1	1	1
Psychiatric nurses		2	2	2	2
Social educators		1	1		
Social workers		2	1		1
Nursing assistants		1			
Peer specialists		1		1	

### Data collection

This qualitative study draws on different data sources, including case reviews, focus group interviews, and individual interviews. From November 2013 to August 2015, after the initial patient interviews [44], the first author (HKS) read the patients’ case files, once before and nearly one year after interviewing the RCs. One year after the individual interviews, HKS conducted focus-group interviews, assisted by co-moderators with long experience from treatment and research, to explore the RCs’ considerations and the ACT providers’ experiences with the follow-up of patients enrolled in ACT and subjected to CTOs.

The individual interviews and the focus-group interviews were conducted using a thematic interview guide developed jointly by the authors, based on a literature review (Additional file 1 and Additional file 2). Even if we had data about the 15 patients’ views and experiences with the ACT teams’ enactment of CTOs, we only referred to the patients’ CTO status in the provider interviews. All the interviews started with open questions about their experiences with the CTO follow-up responsibility, and how the CTO responsibility was organized. We also asked the participants to use some of the specific cases to describe the content of the CTO and its clinical implications. We opened up with phrases like, “When we interviewed (X) he was placed under a CTO, which later was removed, but is now back on a CTO. Have you had recent team discussions about the length of the CTOs and the CTO follow-up responsibility?” Even if we used case examples, participants also described other CTO cases.

We asked the focus group participants about the strategies applied to develop supportive relationships with the patients and their experiences with CTOs. The topic guide was based on previous patient interviews, case files, and the interviews with the decision makers. All the interviews were conducted within the ACT team’s facilities. The individual interviews lasted 55–110 min. The focus-group interviews lasted 120–130 min, where the co-moderator was invited in to ask for more details and to summarize. The interviews were recorded and transcribed verbatim.

### Analysis

We utilized a modified grounded theory approach informed by a constructivist and interpretative framework [45]. The approach is a recognized method for investigating phenomena where little prior knowledge exists. An iterative process of data collection was used to develop a conceptual understanding of the ACT providers’ and the RCs’ experiences with CTOs based on the categories grounded in the data [45]. The interviews were consecutively thematically coded, to identify meaning units in the transcribed text. Subsequently, we used the same procedures for the focus group interviews. After the initial coding, all the interviews and the memos were read through, to compare the most frequently used codes and develop more focused codes. The most central codes were clustered in theoretically linked themes, which allowed us to develop categories and sub-categories that were linked together, based on their properties and dimensions (focused coding). Subsequently, categories were linked together (theoretical coding). Focused coding was done manually, and thereafter NVivo software [46] was used to get a better overview of the data. The process of constant comparative analysis was continued with the help of the software until no additional new observations emerged. Memo writing was used through the process, to increase the level of abstraction and to aid in the development of the categories.

### Ethical considerations

This study was approved by the Regional Committee for Medical and Health Research Ethics (Case number 2010/1196a). The team leaders were given written information about the study before consenting to participate. The team members were informed about the study and that participation was voluntary prior to choosing to participate in the interviews. In connection with the patient study [44], we asked for permission to use case files and to talk with the ACT providers and the RCs about their case. All data were kept confidential and stored in unidentifiable form.

## Results

The participants presented the ACT model’s structural components as a window of opportunity to approach the patients’ needs. According to the participants, CTOs were mainly used to ensure medication adherence and monitoring, but was also presented as a safety net for calculated risk-taking within the context and opportunities for engagement and practical support provided by the ACT model. CTO decision-making was described as a complex procedure, and the ACT providers’ commitment to establish supportive relationships was a central topic in their accounts. Three categories leading to the main theme of ‘Responsibility with conflicting priorities’ were identified: ‘Conflicting priorities: Treatment and autonomy’, ‘ACT and providing practical support and comprehensive services’ and ‘ACT and building rapport’.

### Conflicting priorities: Treatment and autonomy

The successful use of CTOs was presented as resource-demanding and involved conflicting values and considerations. Respecting patients’ autonomy while also meeting their clinical duty to provide treatment was often a difficult balance to strike. As one psychiatrist said:

“*Having people under coercion goes against the idea of running your own life, being allowed to make your own choices, make mistakes, learn from your mistakes ... it’s a difficult balancing act.”*

Even if the team frequently had to remind some patients about the need to take medications to stay well and sometimes use police assistance to bring the patients to a treatment ward, most patients adjusted to the “rules” and “conditions” of the CTO. As one ACT provider said:

“*There is a threat of being readmitted. They know that if they do not collaborate they might be admitted, so in one way we become a necessary evil.*”

Many ACT providers described dilemmas and conflicts regarding medications. Both the inherent asymmetrical power balance and the clinicians’ dual role in combining care with compulsion involved ethical and practical difficulties. As one ACT provider said:

“*It’s difficult. We talk a lot about it, especially those of us who travel around giving depot injections. We face a lot of resistance to medication, there’s a lot of coercion involved. We talk about it and reflect on it in the mornings, how to do it in the best possible way and what we could do differently, we do try. I have to speak for myself. I must say that I sometimes feel uneasy, because I don’t always think this helps.”*

The psychiatrists emphasized the importance of antipsychotic treatment to prevent psychotic episodes and in some cases, often regarding patients with concurrent substance abuse; long- term use of CTOs was justified as the least restrictive solution. As one psychiatrist said:

“*The reason why I don’t dare to remove the CTO is the patients’ rapid symptom exacerbation associated with substance abuse.*”

As many psychiatrists claimed, as a multidisciplinary team they could be much more responsive to the patients’ treatment needs and wishes, and in some cases they would use the CTO as a safety net for a limited time period, to reduce or take patients off medications. One psychiatrist said:

*“The art is to find the right maintenance dose and eventually, if it is justifiable to let people become psychotic again, I might terminate the CTO, or let people discontinue medications with the legal framework in place.”*

It was often seen as helpful to try to establish a common understanding with patients about the need for medications. A focus on patients’ possible choices and possibilities, rather than on the disagreements was also seen as helpful. Even if the team could not compel patients to take medications unless a valid medication order was in place, the ACT providers would often intervene early to try to convince the patients about the need to take medications to stay well. As one ACT provider said:

*“Patients on CTOs do not get the opportunity to stop medications for a longer period if that is unadvisable.”*

The teams were often concerned about patients’ substance use, previous history of violence associated with treatment discontinuation, and the early discharge from the Emergency Wards of patients suffering from substance induced psychosis. In such cases the ACT providers felt powerless despite the CTO:

“*With (X) we experience powerlessness about what we believe is good for the patient and what we can actually do.*”

According to many ACT providers, there was a fine line between educating and convincing patients about the need to take medication and what would amount to pressuring them. For example, it was not always clear when reminders about previous illness episodes might be perceived of as threats. The Supervisory Commission had, at the time of data collection, recently given instructions to one of the teams that CTOs did not authorize threats or persuasive communication about hospital admissions. The ACT providers we interviewed acknowledged that the CTO scheme did not allow threats of hospitalization. As long as a CMO was not in place, the patient had the final word about depot injections, even if that might imply adverse outcomes for the patient.

### ACT providing practical support and comprehensive services

CTOs were used to secure treatment adherence, sometimes referred to as a ‘platform for other interventions’. A narrow medication perspective was described as inadequate. Supporting the patients to better manage their symptoms, so that they gradually could take more responsibility for their own lives was deemed equally important. ‘To support people to reconnect with life’ was how several ACT providers described the ACT team’s vision, and many said that the ACT model enabled them to take a wide approach in their work with patients. As one ACT provider said:

*“I’m thinking about what you’re saying here, helping people take the bus and so on. We get people who’ve been in hospital for a long time, who’ve been institutionalized and then we have to start from scratch with many of them and help them get back to life after being in an institution for a long time.”*

A range of factors*,* everyday skills training, securing access to welfare benefits and safe housing, helping building social networks, participating in activities and developing a crisis plan or initiating regular voluntary inpatient arrangements were important to stabilize the patients’ lives. Some saw a role for CTOs in order to achieve the ACT aims. As a psychiatrist said:

“Generally speaking, I often regard the CTO as a mechanism to ensure the social relationships and to establish necessary structures.”

The same psychiatrist followed up by saying that many patients need comprehensive and integrated services, and the team’s opportunity to provide a range of different interventions was important:

*“That we can provide a variety of approaches simultaneously. Everything from providing a guardian, helping with the Economy, Welfare and Labor Administration, medical treatment and soccer training session and dish washing together. And drug addiction treatment, motivational interviewing and bus training simultaneously, simply invaluable.”*

Providing comprehensive support and having frequent contact with patients was perceived as creating opportunities to address the patients’ motivation to engage in treatment. As one psychiatrist said:

*“Because we have established a relationship, we can follow up very ill people, also when they are acutely ill, without the need to rely on coercion. Even if psychotic patients don’t always think we have good intentions, with 2-3 encounters every week, also during crises, we have a better opportunity to work with the patients’ motivation, which is difficult if you meet the patient once or twice a month at an outpatient clinic.”*

Many participants described a gradual change in the teams’ treatment approaches. For example by adopting the ACT principle of regular critical team reflections. Others pointed out that having a peer specialist in the teams had been important to improve practice:

*“Since I started in ACT 5 years ago there has been a change in our mindset. Having a peer specialist broadened our focus, to see that medication is not the only effective ‘medicine’ in a person’s life. We focus more on employment, different activities, not least a place to live, economy, all the factors that bring safety to a person’s life.”*

The participants emphasized the many nuances involved in the content and enforcement of CTOs, and that shared problem solving and critical team discussions were essential. The participants experienced that many patients on CTOs gradually acknowledged the advantages of ACT. As one of the peer specialists said:

*“Many patients have negative experiences with the support services and that’s probably the main reason why the ACT team was created. To realize that these are people who can also take care of other tasks or areas of life, I think that’s a positive experience for many patients.”*

### ACT and building rapport

Because many patients had negative prior experiences with mental health services, building rapport with the patients was described as the most important step to promote treatment acceptance. Even if many participants underpinned that coercion potentially could impede the therapeutic alliance, CTOs were often described as a necessary mechanism to create relationships that could promote stability and safe community living. As one of the psychiatrists said:

*“We have a number of patients where we can see that the CTO has made them better, that it’s basically meant that we’ve got into a treatment position. Saying that we’re here to stay even if you reject us, where they’ve eventually got a relationship with someone in the team precisely because they’ve been on a CTO. The obligations you have give you closer contact and you get a better general idea and you can provide a different approach than we could have without the CTO.”*

The participants emphasized that being part of a multidisciplinary team with shared responsibility that provided flexible follow-up services represented a major improvement from traditional outpatient services*.* The teams’ shared agreement to prioritize the initial engagement process and to spend time with patients across different social arenas was crucial in order to build trust and rapport. The ACT providers’ commitment to establish supportive relationships encompassed something akin to a “we do what it takes” approach. Moving beyond their typical professional roles was in many cases described as an important engagement strategy, to provide continued offers acceptable to patients. The participants emphasized that they used the same engagement strategies with patients on CTOs as with patients not on CTOs. They said that listening carefully and responding to the patients’ wishes was crucial. As one of the ACT providers said:

*“We use the same approaches as we use with patients not on CTOs. We want them to be involved in treatment planning decisions as far as possible, and we try to facilitate, listen to them if they have wishes, if things should be different. I think we treat all the patients with respect, also when they are subject to CTOs.”*

One strategy often mentioned was to let the social worker or the peer specialist, who were not directly involved in medication administration establish the initial contact with the patient, and to gradually introduce other staff members. Phrases like “good cop, bad cop” were used to describe this difference in role functions. Another important strategy was that one team member established contact while the patient was still admitted to the ward, sometimes to create a position as the person the patient ‘hated the least’.

Engaging reluctant patients was described as laborious work; respect and empathy as well as communication skills, patience, and persistence were crucial success factors. Many ACT providers emphasized they often knew their patients well, and when they managed to establish a dialogue about a patient’s wishes and difficulties, they could start to take more ‘risks’ by allowing increased patient autonomy. As one ACT provider said*:*

*“Most important, when that communication is well established, then we can take more risks with our patients”.*

Also, the psychiatrists emphasized that the CTO decision responsibility in ACT was quite different from the traditional office-based services at the CMHC. The ACT model’s team approach, the opportunity to provide regular in-vivo assessments and the daily team meetings increased their confidence in working with this patient group. One psychiatrist explained that without the ACT context the work would have been much more difficult:

“*Then I would have come from outside, I would have been unsure about my assessments in a completely different way from how I feel now.”*

As well as considering the patients’ symptom severity, concurrent substance abuse and previous illness episodes, the patients’ illness insight and the patients’ collaboration and motivation for treatment were seen as important factors in CTO decisions. Furthermore, as several of the psychiatrists pointed out, few guidelines or decision aids exist in this field, and CTO discontinuations were often presented as tough decisions to make.

The ACT approach includes continuous team discussions about what should be in place to safely terminate a CTO. This allowed for flexibility, calculated risk taking and voluntary solutions. Several psychiatrists reported that that they felt the follow-up was safer and better controlled due to the ACT team’s comprehensive and assertive strategy. Also, the ACT approach allowed for reduced coercion and increased patient autonomy. As one psychiatrist said:

*“The ACT team is a means of reducing the use of coercion because if you see the patient as rarely as it often happens when you’re in an outpatient clinic you’re more unsure. I think it’s more convenient to have them under coercion than to have them in voluntary treatment. There are some key practical things that don’t need to be so important in our case. We can juggle more and feel it doesn’t matter so much. We’re trying voluntary care because we have a good alliance with the patient, and if it doesn’t work, we have a framework around us that enables us to cope with that.”*

## Discussion

A main finding in this study was that the participants believed the CTOs were sometimes necessary in order to provide continued treatment. However, the follow-up of CTO patients in the ACT setting involved conflicting priorities. Patients enrolled in ACT often have low functioning and concurrent substance abuse, and the ACT approach was considered as helpful in that it afforded comprehensive and patient-centered support and opportunities to build rapport. The long-term use of CTOs might not be in line with the ACT model’s focus on recovery and person-centered care. However, for some patients, the participants saw few alternatives to CTOs as a long-term safety measure to prevent relapse and possible harm to the patients themselves or other people.

### Moving toward patient-centered approaches

Many patients subject to CTOs had a recent history of treatment discontinuation, and as other studies show, CTOs were described as a clinical tool to promote treatment adherence to stabilize the patients’ condition and to prevent hospital re-admission [14, 15, 20–23]. The participants emphasized the importance of communication and of facilitating patient cooperation. Seeing the patients frequently and assisting them with everyday activities was considered pivotal. Patients enrolled in ACT were often hard-to-reach, and compared to other treatment contexts, the boundary between voluntary treatment and coercion seemed more blurred. The engagement process often involved careful balancing between collaborative approaches to establish contact and being intrusive, and as other studies show, building trust and rapport with the patients was described as the most important step to promote treatment acceptance [47–49].

CTO decisions were described as challenging and complex. The inherent power imbalance between clinicians and patients and the clinicians’ conflicting priorities were perceived as problematic for the therapeutic relationship. Despite many patients’ adjustment to the CTO conditions, conflicts regarding medications were ubiquitous. It was not always clear when reminding patients about previous illness episodes and the risk of not taking medication should be considered as verbal guidance, persuasion, or threats. However, the participants emphasized that when patients were placed on CTOs, the ACT approach could address non-adherence and relapse at an earlier stage than traditional services and more easily offer patients choices and negotiate treatments during crises.

### Addressing clinical uncertainty through shared responsibility

The psychiatrists in our study distinguished between an insider and an outsider perspective to describe their dual responsibilities in ACT, where the ACT model’s structured approach was described as an important quality improvement. Shared case-load responsibility and daily team meetings increased the psychiatrists’ confidence, and as many participants pointed out, the team’s comprehensive follow-up services promoted flexibility and more voluntary solutions. Several of the psychiatrists felt more secure in their decisions to reduce the medication dose or remove the CTO when they could discuss these issues in their teams. As other studies have shown [13, 23, 24], clinicians are concerned about factors such as lack of insight and risk of relapse when making decisions about CTOs. Some prior studies have found that the duration of CTOs seems to depend on individual decision maker’s judgments [14, 22, 23]. The ACT approach, with frequent patient contact, the building of rapport and trust and team-based decisions, is likely to afford clinicians more security and leeway in their decision-making processes. On the other hand, engaging reluctant patients was described as laborious work, and as many participants in our study indicated, lack of time and available treatment resources could lead to an overly defensive CTO practice.

### Addressing adherence through assertiveness

During the last the last 15 years the ACT model has had a clear focus on integrating the promotion of autonomy, client choice, collaborating treatment planning and self-directed care [40, 50]. The ACT providers and the RCs in our study highlighted that even if many patients disagreed about the need for medication, many patients gradually acknowledged the benefits of ACT, such as help with obtaining safe housing and welfare benefits. Studies have suggested that services that emphasize empowerment and recovery can improve outcomes [50, 51]. Although the ACT model has a strong emphasis on recovery, medication administration and monitoring are cornerstones of the model. Some studies have found that ACT providers often use targeted efforts to encourage adherence to medication [52–54]. Other studies show that some Assertive Outreach Teams have reported using less restrictive practices and more varied and flexible approaches than less intensive Community Mental Health Teams [55–57]. Interestingly, a prior study found that patients subject to CTOs in Norwegian ACT teams were more satisfied than patients without CTOs [58]. Our respondents’ overall positive view of ACT stands in contrast to some other studies describing concerns relating to paternalism, rights to privacy and self-determination and human rights [59–61].

### Assertiveness as a means to increase autonomy

The participants stated that mandated community treatment was justified when it was seen as necessary in order to protect patients or others from dangerous or threatening behavior. However, balancing effective care and autonomy was difficult [62]. Using overly paternalistic approaches was seen as a pitfall, as was underestimating a patient’s vulnerability, impaired decision making and willingness to take part in treatment decisions. Participants’ descriptions of patients’ self-efficacy and agency resonate with a relational understanding, where autonomy is achieved and exercised in the context of supportive relationships and available opportunities in the community [63–65]. Studies suggest that many patients experience CTOs to be disempowering, usually because the patients experience a one-sided focus on medications and not being sufficiently involved in treatment decisions [14, 16–19]. Focusing on procedural justice, which refers to the patients’ perceptions of fairness and being treated with respect, can be important in mitigating experiences of coercion [66]. Our respondents discussed the importance of building trust and involving patients in treatment planning decisions. A long-term commitment was seen as necessary in order to improve the patients’ life conditions and thereby help them to gradually take more control of their own lives [64, 65].

These findings differ somewhat from those in Lawn et al.’s study [16], where providers were drawn into a coercive role as their first response to patients who resisted their views of what was needed. Lawn et al. [16] found that providers did not engage these patients in a meaningful dialogue about their personal experiences of CTOs, and that their practice was predominantly focused on risk management and compliance. Many participants in our study referred to CTOs as a safety net to facilitate medication reduction, especially with patients with a history of rapid relapse or serious illness episodes. It was important to try to establish a common understanding about the need for medications and the patients’ possible choices and opportunities, especially if it was uncertain whether the medication was effective [67]. Some ACT providers described a reorientation in the ACT team’s medication approach as a learning process, from a deficit-oriented focus (i.e. on symptoms and medication) to a more person-centered and recovery-oriented approach. Furthermore, to sit down as a team to critically reflect on ethical dilemmas and the course of action, also to ‘keep each other in check’, was in line with what other studies have found to be important to improve practice [48, 49].

### ACT and levels of coercion

Swartz and colleagues emphasized that a court order might have an effect on both patients and the service system [28]. In some contexts, patients on CTOs may be given priority to ACT treatment. Our participants emphasized that they used the same approach for patients on or off CTOs. It was not required that patients were on CTOs in order to receive ACT or any other services. The participants in our study highlighted that the impact of CTOs depended on how they were implemented and the services that were provided [68]. From September 2017, a capacity criterion has been introduced in an amendment to the Norwegian MHA so that patients with decision-making capacity no longer can be treated involuntarily unless they or their surroundings are in acute danger. It is unclear how the recent changes will impact clinicians’ decision-making processes regarding CTOs. The intention is to reduce coercion. However, some have pointed out that this has not consistently been the case in other countries where legal changes have been made in an effort to reduce coercion [69–73]. Some prior research has suggested that CTOs do not improve patient outcomes [1]. Moreover, it is unclear if any improved outcomes may be attributable to enhanced community services rather than to their compulsory nature. Increasing resources and improving the quality of services might lead to reduced coercion [74]. Nevertheless, with changes in the Norwegian MHA, some commonly accepted benchmarks for what constitutes good practice and acceptable risk seems required.

### Limitations

This study has several limitations. The greatest limitation is the small number of teams included. The sample consisted of four ACT teams, so the results may not be widely generalizable. The teams were sampled purposively in order to include teams with different CTO rates and that also organized the CTO responsibility differently. In this way, we hoped to capture variations in the ACT providers’ and the RCs’ experiences with CTOs. Most ACT providers had participated in ACT training and workshops, supported by a Norwegian ACT handbook [75] and fidelity assessments after 12 and 30 months. To avoid a one-sided initial enthusiasm for the ACT models’ flexible and integrated care approach, we specified that the selected focus-group participants should have at least two years’ experience. However, not all of the 20 staff members were as experienced as we had hoped.

## Conclusions

The results from this study highlight the challenges and complexities involved in CTO decisions in an ACT context. Although long term use of CTOs may conflict with the ACT model’s focus on recovery and person-centered care, for some of the patients, the participants saw few alternatives to CTOs as a long-term safety measure to prevent relapse and possible harm to self or others. The participants highlighted the team approach, and the importance of frequently meeting the patients on different social arenas. This gave them insight into the everyday lives of the patients and a greater sense of security when taking patients off CTOs. Despite new regulatory changes clinicians are still faced with the dilemma of balancing coercion and autonomy. Future research should examine ACT providers and clients’ perspectives of CTOs over time.

## Additional files

Additional file 1: Interview/discussion guide for focus groups. (DOCX 81 kb)

Additional file 2: Interview/discussion guide for Responsible Clinicians. (DOCX 16 kb)

## Abbreviations

ACT: Assertive community treatment
CMHC: Community Mental Health Centre
CMO: Compulsory medication order
CTO: Community treatment order
MHA: Mental Health Care Act
RC: Responsible Clinician
SC: Supervisory Commission
