# Supplementary material for: Responsibilities with conflicting priorities: a qualitative study of ACT providers’ experiences with community treatment orders
Source: BMC Health Serv Res. 2018 Apr 18;18:290. doi: 10.1186/s12913-018-3097-7 (PMC5907185; doi:10.1186/s12913-018-3097-7)
Supplement: Supplementary file 1 — Interview/discussion guide for focus groups. (DOCX 81 kb) [file 12913_2018_3097_MOESM1_ESM.docx]

**Supplementary file 1: Interview/discussion guide for focus groups**

**Key themes**

1. How do you consider ACT compared to traditional outpatient services?

2. How do you start to establish contact and engage newly enrolled patients?

- What about patients on CTOs? (strategies)

3. How is the CTO responsibility organized?

4. To what extent, and in which ways do CTOs influence your work? (and the therapeutic relationship, strategies)

5. What is your role and to what extent is the team involved in discussions/decisons regarding CTOs?

6. What is the content of CTOs?

7. How do you manage treatment refusal (medication, appointments)

8. What are your main concerns in CTO cases (priorities)?

9. What are the most important benefits/disadvantages of CTOs?

9. Do you have spcific criteria or checklists regarding CTOs? (treatment compliance, appointments, symptom stability)?

10. Who are responsible for providing information about CTO decisons to patients and relatives?

**Specific CTO cases**

You have been involved in treatment planning decisions for XX

What have been the most important considerations in that case?

-To what extent has voluntary treatment been tried?

-Risk and the threshold for readmssion?

-Can you describe what happened the last time he/she was admitted?

-In which circumstances could the CTO be terminated?
