# Supplementary material for: Responsibilities with conflicting priorities: a qualitative study of ACT providers’ experiences with community treatment orders
Source: BMC Health Serv Res. 2018 Apr 18;18:290. doi: 10.1186/s12913-018-3097-7 (PMC5907185; doi:10.1186/s12913-018-3097-7)
Supplement: Supplementary file 2 — Interview/discussion guide for Responsible Clinicians. (DOCX 16 kb) [file 12913_2018_3097_MOESM2_ESM.docx]

**Supplementary file 2: Interview/discussion guide for Responsible Clinicians**

**Key themes**

1. How will you describe ACT compared to traditional outpatient services?

2. How will you describe your role/responsibility in ACT?

3. How is the CTO responsibility organized?

4. What is your role and to what extent is the team/other clinicians/relatives involved in CTO decisions?

5. What is the content of CTOs?

6. How do you manage treatment refusal (medication, appointments)?

7. What are your main concerns in CTO cases (priorities)?

8. Do you have specific criteria in CTO decisons? (treatment compliance. appointments, symptom stability, dangerousness)?

9. If patients refuse to take medication and their mental health condition deteriorates, what is the threshold for readmission?

10. What are the most important benefits/disadvantages with CTOs?

11. Do you consider CTOs primary as a risk managment tool (advantage for society) or as a tool to improve the patients' well being and recovery?

12. Who is responsible for providing information about CTO decisons to patients and relatives?

**Specific CTO cases**

You have been involved in treatment planning decisions for XX

-What have been the most important considerations in that case?

-To what extent has voluntary treatment been tried?

-Risk and the threshold for readmssion?

-Can you describe what happened the last time he/she was admitted?

-In which circumstances could the CTO be terminated?
